# Simian Virus Large T Antigen Interacts with the N-Terminal Domain of the 70 kD Subunit of Replication Protein A in the Same Mode as Multiple DNA Damage Response Factors

**DOI:** 10.1371/journal.pone.0116093

**Published:** 2015-02-23

**Authors:** Boting Ning, Michael D. Feldkamp, David Cortez, Walter J. Chazin, Katherine L. Friedman, Ellen Fanning

**Affiliations:** 1 Department of Biological Sciences, Vanderbilt University, Nashville, Tennessee, United States of America; 2 Departments of Biochemistry, Chemistry, and Center for Structural Biology, Vanderbilt University, Nashville, Tennessee, United States of America; 3 Department of Biochemistry, Vanderbilt University Medical School, Nashville, Tennessee, United States of America; Saint Louis University, UNITED STATES

## Abstract

Simian virus 40 (SV40) serves as an important model organism for studying eukaryotic DNA replication. Its helicase, Large T-antigen (Tag), is a multi-functional protein that interacts with multiple host proteins, including the ubiquitous ssDNA binding protein Replication Protein A (RPA). Tag recruits RPA, actively loads it onto the unwound DNA, and together they promote priming of the template. Although interactions of Tag with RPA have been mapped, no interaction between Tag and the N-terminal protein interaction domain of the RPA 70kDa subunit (RPA70N) has been reported. Here we provide evidence of direct physical interaction of Tag with RPA70N and map the binding sites using a series of pull-down and mutational experiments. In addition, a monoclonal anti-Tag antibody, the epitope of which overlaps with the binding site, blocks the binding of Tag to RPA70N. We use NMR chemical shift perturbation analysis to show that Tag uses the same basic cleft in RPA70N as multiple of DNA damage response proteins. Mutations in the binding sites of both RPA70N and Tag demonstrate that specific charge reversal substitutions in either binding partner strongly diminish the interaction. These results expand the known repertoire of contacts between Tag and RPA, which mediate the many critical roles of Tag in viral replication.

## Introduction

DNA replication is an essential and highly regulated event in all organisms, requiring faithful duplication of the genome during each cell division cycle. Simian Virus 40 (SV40) provides a powerful model for eukaryotic replication since the ~5 kb viral genome is replicated almost entirely by the host replication machinery [1].

**Fig 1:**
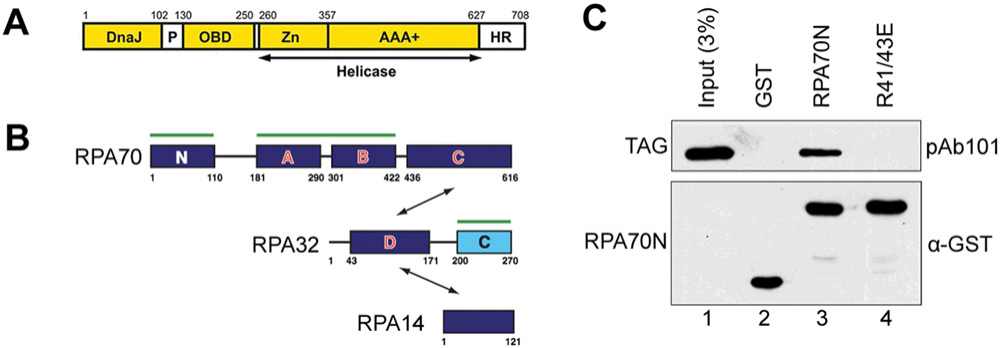
RPA70N binds to Tag. **A**. Depiction of Tag functional domains and Tag fragments utilized in this study. **B**. Depiction of RPA subunits. Amino acid residues are numbered. DNA binding domains from A to D are shown in red letters. Protein binding surfaces are labeled with green lines. Double-headed arrows are used to show interactions between RPA subunits. **C**. Western blot of GST pull-down assays between RPA70N and full-length Tag. Purified GST-RPA70N WT and R41/43E were incubated with glutathione beads for 1 hour, and then incubated with Tag purified as described in Materials and Methods. Bound proteins were eluted, resolved by SDS-PAGE, and immunoblotted with GST antibodies or pAb101 (recognizes Tag) as indicated.

Remarkably, the only viral protein required for SV40 DNA replication is the multifunctional Large Tumor Antigen (Tag) [1]. Early during infection, Tag inhibits p53 and the Retinoblastoma family of tumor suppressors to force the host cell to remain in S phase, thus promoting a cellular environment that is favorable for viral replication [2]. Tag also serves as the DNA replication initiator and replicative helicase [3, 4]. Tag has four functional domains: a J-domain at the N-terminus that functions as a DnaJ molecular chaperone to inhibit Rb protein function, the origin DNA binding domain (OBD) between residues 131 and 259, a helicase domain containing a zinc binding motif and AAA+ ATPase motor between residues 260 and 627, and a disordered host range determinant domain near the C-terminus (Fig. 1A) [5–8]. Residues 102–130 comprise an extended linker that includes an LXCXE motif required for interaction with Rb proteins, a nuclear localization signal, and several phosphorylation sites that affect multiple Tag functions [9, 10].

During the initiation of viral DNA replication, Tag interacts with four GAGGC pentanucleotide repeats located in a central palindrome of the SV40 origin of replication. This central palindrome is flanked by an imperfect palindrome and an AT-rich region [11]. One hexamer of Tag binds to the early-transcript side of the core origin, followed by loading of a second complex to form a head-to-head double hexamer. Similar to the eukaryotic replicative helicase, the Tag hexamers unwind the viral DNA at the fork, enabling bidirectional DNA replication [12, 13]. Tag is not sufficient to support origin initiation *in vitro*, but requires host cell contributions from topoisomerase I, DNA polymerase α/primase, and the single-stranded DNA (ssDNA) binding protein Replication Protein A (RPA) [14]. Multiple interactions between these four proteins are necessary to support productive initiation of DNA replication [15, 16].

RPA is a heterotrimeric protein containing subunits of 70, 32 and 14 kDa, with 6 OB-fold, one winged helix, and one disordered domain and a number of highly flexible linkers (Fig. 1B). Its ssDNA binding activity is mediated by four OB-fold domains, A, B, and C in RPA70 and D in RPA32 [17]. RPA initially binds 8–10 nts of ssDNA with the tandem RPA70AB domains, then transitions its architecture so that the trimer core containing the 70C and 32D domains (along with RPA14) can engage, leading to the final high affinity 24–30 binding mode [18]. It has been previously postulated that at the initiation of the priming step of SV40 replication, Tag recruits RPA though an interaction with the winged helix domain RPA32C [19]. Tag is then thought to facilitate priming by DNA polymerase α-primase (pol-prim) in part through an interaction with the RPA70AB that favors the lower affinity RPA architecture, freeing up the template for binding by pol-prim [20].

Direct physical interactions between Tag and RPA have been mapped to RPA70AB and RPA32C domains, both of which contact the Tag OBD [20, 21]. We had previously observed that, in addition to a number of DNA damage response proteins (e.g. ATRIP, RAD9, MRE11, p53), the RPA70N protein interaction domain interacts with human DNA helicase B (HDHB) [22–25]. Given interaction with this helicase, we hypothesized that Tag may also interact with RPA through the RPA70N domain. In this report, we show that there is a direct physical interaction between Tag and the RPA70N domain. We go on to map the interaction to a sequence within the Tag J-OBD linker and the RPA70N basic cleft that is used as a binding site by all previously characterized RPA70N binding partners.

## Materials and Methods

### Purification of GST-tagged proteins

Rosetta 2 cells (EMD Millipore, Darmstadt, Germany) expressing glutathione S-transferase (GST)-tagged recombinant proteins were resuspended in lysis buffer (1X phosphate buffered saline (PBS), 1% Triton X-100, 7 mM MgCl_2_, 1 mM ethylenediaminetetraacetic acid (EDTA), 1 mM dithiothreitol (DTT), 0.2 mM phenylmethylsulfonyl fluoride (PMSF), 1 mM benzamidine, 5 nM aprotinin, 0.01 μM leupeptin) and sonicated two times for 30 seconds followed by one time for 20 seconds (Branson 250, duty cycle 90%, output control 8). Sonicated lysate was centrifuged for 25 minutes at 20,000 rpm at 4°C. The resulting supernatant was combined with glutathione agarose beads (Thermo, Rockford, IL) and incubated for 3 hours at 4°C. Following incubation, beads were washed two times with 20 bed volumes of wash buffer 1 (1X PBS, 1% Triton X-100, 7 mM MgCl_2_) and one time with 20 bed volumes of wash buffer 2 (1X PBS, 7 mM MgCl_2_). The beads were transferred to a 10 ml polyprep column (Biorad) using 10 ml of wash buffer 2. Protein was eluted four times with 1 ml elution buffer 1 (50 mM Tris-Cl pH 7.9, 10 mM glutathione) and two times with 1 ml of elution buffer 2 (50 mM Tris-Cl pH 7.9, 25 mM glutathione). Proteins were dialyzed in dialysis buffer (25 mM Tris-Cl pH 7.5, 25 mM KCl, 0.5 mM EDTA and 10 mM imidazole). The full-length Tag and Tag(84–130) proteins used in isothermal titration calorimetry (ITC) and nuclear magnetic resonance (NMR) studies were treated with H3C PreScission protease (Genscript, Piscataway, NJ) and the cleaved tags were removed by passage through a glutathione bead column as described above.

### Purification of His-tagged proteins

Purification of 6-histidine and maltose binding protein (His-MBP)-tagged proteins followed the same basic protocol as purification of GST-tagged proteins, with the following changes. The pre-chilled lysis buffer consisted of 25 mM Tris pH 8, 150 mM NaCl, 0.5 mM EDTA, 10 mM imidazole, 1% Triton X-100, 5 nM aprotinin, 200 μM PMSF, 1 mM benzamidine, 0.01 μM leupeptin. Nickel-Nitrilotriacetic acid (Ni-NTA) resin (Qiagen) was washed in 25 mM Tris pH 8, 300 mM NaCl, 0.5 mM EDTA, 10 mM imidazole. Three buffers were used for stepwise elution: 25 mM Tris pH 8, 150 mM NaCl, 0.5 mM EDTA with addition of 40 mM imidazole, 80 mM imidazole, or 250 mM imidazole. Eluted fractions were visualized by Coomassie stain and selected fractions were pooled for overnight dialysis in 2 L buffer (25 mM Tris pH 8, 25 mM KCl, 0.5 mM EDTA) at 4°C.

### GST pull-down assays

Glutathione agarose beads (Thermo, Rockford, IL) were equilibrated with 10 bed volumes of wwwater and 2 x 10 bed volumes of binding buffer (30 mM HEPES KOH pH 7.8, 10 mM KCl, 7 mM MgCl_2_, 10 μM ZnCl_2_, 2% non-fat dry milk). 20 μl GST-fusion proteins (4 μM) were added to the beads in 200 μl binding buffer and rotated at 4°C for 1 hour. Beads were centrifuged at 2,000 rpm for 2 minutes at 4°C. The supernatant was discarded and beads were washed with 500 μl binding buffer. Following removal of the supernatant, 1.14 nmol of the prey protein was added in 200 μl binding buffer and the mixture was rotated at 4°C for 1 hour. The beads were washed once with binding buffer and 3 times with wash buffer (30 mM HEPES KOH pH 7.8, 75 mM KCl, 7 mM MgCl_2_, 10 uM ZnCl_2_, 0.25% myo-inositol, 0.01% NP-40). Following the last wash, the beads were re-suspended in 20 μl of sample loading buffer, heated to 80°C for 10 minutes, and separated by SDS-PAGE prior to immunoblotting.

In the pAb416 (recognizes Tag(84–130)) blocking assay, prior to a standard GST pull-down, full-length Tag was incubated with either IgG (nonimmune rat) or pAb416 at 1:1 molar ratio. This mixture was incubated with GST-RPA70N bound to glutathione beads followed by separation of the reaction mixture by SDS-PAGE for immunoblotting. For epitope mapping assays, 50 nmol of GST-Tag fragments (4–260, 260–627, 1–130, 131–259, 1–84) were separated by 12.5% SDS-PAGE and transferred to membrane for immunoblotting.

### Antibodies

Primary antibodies used for Western blotting were: mouse anti-His at 1:5,000 (Genscript, Piscataway, NJ), rabbit anti-GST at 1:40,000 (Invitrogen, Carlsbad, CA), monoclonal anti-Tag pAb101 and pAb416 at 1:10,000 [26, 27]. Nonimmune rat IgG was purchased from Jackson ImmunoResearch. The proteins were visualized with Jackson Research anti-mouse or anti-rabbit horseradish peroxidase at 1:10,000 or 1:5,000. Chemiluminescence (ECL, PerkinElmer Life Sciences) was used visualize the protein by exposure to film.

### Cloning of Tag fragments and mutants

Primers for site-directed mutagenesis of SV40 Tag were: 1R forward: 5’ CGT CGC TGG GAG CAG TGG TGG AAT GCC TTT AAT G 3′ and reverse: 5’ CCC AGC GAC GAG TTC CAT AGG TTG GGG ATC CAC TAG 3’; 1A forward: 5’ AAC TGC CGC ATG GGA GCA GTG GTG GAA TGC CTT TAA TG 3’ and reverse: 5’ TGC GGC AGT TCC ATA GGT TGG GGA TCC ACT AGT TCT AG 3’; 2R forward 5’ TAA TCG TCG CAA CCT GTT TTG CTC AGA AGA AAT GCC ATC 3’ and reverse 5’ GCG ACG ATT AAA GGC ATT CCA CCA CTG CTC CCA TTC 3’; 2A forward: 5’ TAA TGC GGC AAA CCT GTT TTG CTC AGA AGA AAT GCC ATC 3’ and reverse: 5’ TGC CGC ATT AAA GGC ATT CCA CCA CCT GCT CCC ATT C 3’; 3R forward 5’ CAT CTA GTC GTC GTC GTG CTA CTG CTG ACT CTC AAC 3’ and reverse 5’ AGC ACG ACG ACG ACT AGA TGG CAT TTC TTC 3’; 3A forward 5’ CAT CTA GTG CTG CTG CGG CTA CTG CTG ACT CTC AAC 3’ and reverse 5’ CGT CGC TGG GAG CAG TGG TGG AAT GCC TTT AAT G 3’.

A pBluescript vector containing sequence encoding Tag(84–130) between *Eco*RI and *Bam*HI sites was used as template and mutagenesis was conducted using the QuickChange Mutagenesis Kit (Stratagene, La Jolla, CA) with Phusion polymerase (NEB, Ipswich, MA).

### RPA70N Expression and Purification

The production of wild-type RPA70N (aa 1–120 of RPA70) from pET15b vector (Novagen, Darmstadt, Germany) was performed as described previously [28]. ^15^N-enriched RPA70N NMR samples were prepared in a buffer containing 5 mM DTT, 75 mM NaCl, 20 mM Tris and 5% deuterated water (D_2_O) at pH 7.4.

### Isothermal Titration Calorimetry

Tag(84–130) and RPA70N were exchanged into 25 mM Tris-Cl at pH 7.5 and 100 mM NaCl buffer. The binding affinity of Tag(84–130) for RPA70N was measured using a MicroCal VP isothermal titration calorimeter. Titration experiments were performed by 10 μl-injections of 336 μM Tag(84–130) into 60 μM RPA70N in the sample cell. The data were analyzed using Origin software, fitting to a single site binding model using nonlinear least squares analysis. The thermodynamic parameters and binding constants (K_d_) reported are the average of three trials.

### NMR analysis

NMR experiments were performed at 25°C using Bruker Avance 600-MHz NMR spectrometers equipped with a 5-mm single-axis z gradient cryoprobe. Spectra were recorded using band-selective, optimized flip angle short transient, ^1^H, ^15^N-heteronuclear multiple-quantum coherence (SOFAST-HMQC) [29]. 1024 data points were acquired in the direct ^1^H dimension and 96 points in the indirect ^15^N dimension, with a recycle delay of 200 ms. ^15^N-enriched RPA70N was prepared at 26 μM in buffer containing 25 mM Tris-Cl at pH 7.5, 100 mM NaCl and 1 mM DTT. A series of ^15^N-^1^H HSQC spectra were collected at RPA70N/Tag(84–130) ratios of 1:0, 1:0.5. 1:1, 1:2, 1:4, 1:8, 1:12 and 1:16. All spectra were processed with TOPSPIN v1.3 (Bruker, Billerica, MA) and analyzed with Sparky v3.1 (University of California, San Francisco, CA). ^1^H and ^15^N backbone NMR assignments for RPA70N were reported previously [30]. Chemical shift perturbations (Δδ) were calculated using a weighted average of net changes in chemical shift in both ^1^H and ^15^N induced by binding of Tag(84–130) using following equation:

$$\Delta \delta (ppm)\;=\;{\left\{{\left(\Delta^{1}H\right)}^{2}+\;{\left(\Delta^{15}N(0.2)\right)}^{2}\right\}}^{½}$$ 1

## Results and Discussion

### Tag contains a RPA70N binding site between residues 84 and 130

To test whether Tag interacts with RPA70N, purified GST-RPA70N (residues 1–120; Fig. 1A) bound to glutathione beads was mixed with purified full-length Tag (Fig. 1B). After extensive washing, the bound proteins were eluted and examined by immunoblotting with anti-GST and anti-Tag (pAb101) antibodies, using GST alone as a negative control. Full-length Tag interacted with GST-RPA70N but not with GST alone (Fig. 1C, compare lanes 2 and 3).

**Fig 2:**
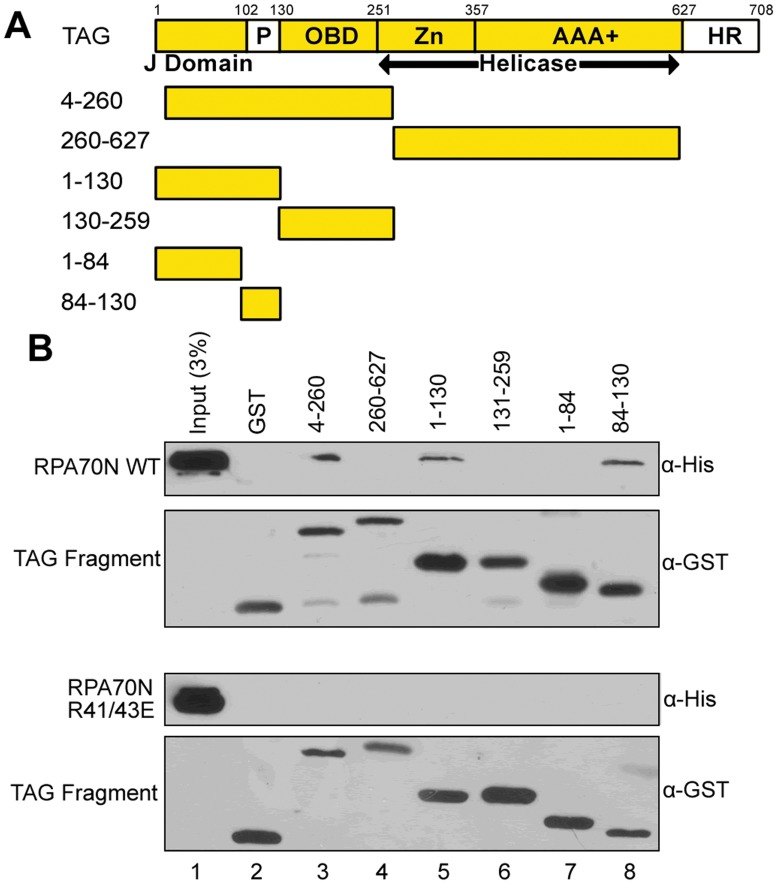
RPA70N interacts with Tag between residues 84 and 130. **A**. Diagram of Tag fragments used for pull-down assay. Amino acid numbers are indicated. **B**. His-tagged RPA70N WT and R41/43E were purified as described in Materials and Methods and added to GST or GST-Tag fragments pre-bound to glutathione beads. Bound proteins were eluted, separated by SDS-PAGE, and immunoblotted with anti-His and anti-GST antibodies as indicated.

To identify the region of Tag that interacts with RPA70N, fragments of Tag fused to GST (Fig. 2A) were purified and tested for interaction with poly-histidine tagged RPA70N (His-RPA70N). Tag fragments 260–627, 131–159 and 1–84 failed to interact with RPA70N (Fig. 2B, top, lanes 4, 6, and 7). However, GST-Tag fragments 4–260, 1–130 and 84–130 interacted with His-RPA70N to similar extents (Fig. 2B, top, lanes 3, 5, and 8), suggesting that RPA70N interacts with Tag between residue 84 and 130. These residues comprise the fourth helix of the Tag J-domain and an adjacent unstructured linker region (Fig. 2A).

**Fig 3:**
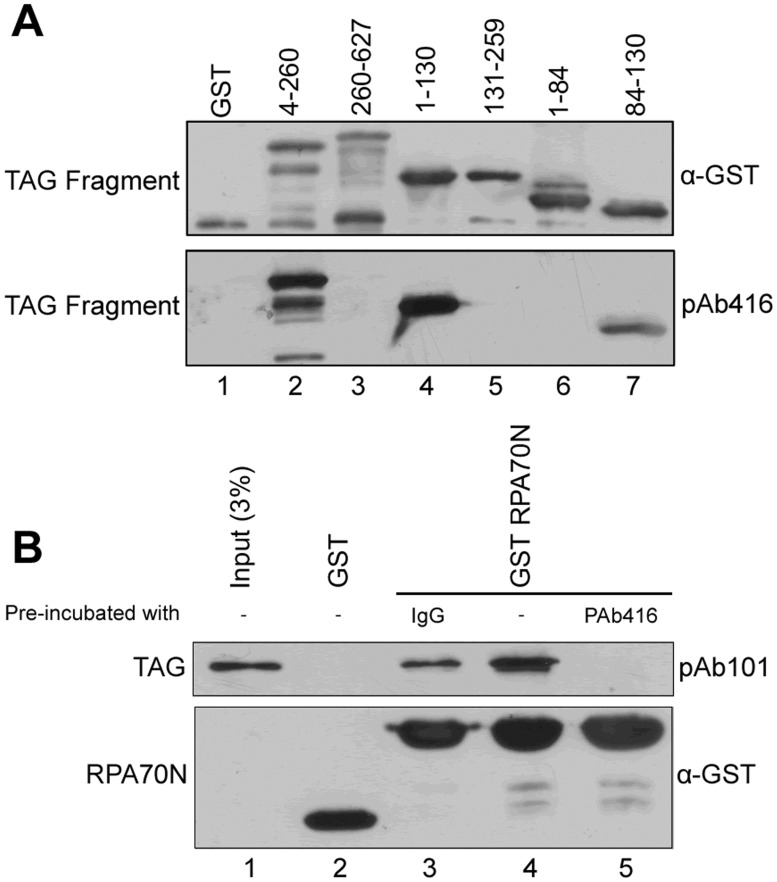
An antibody (pAb416) recognizing Tag(84–130) blocks the interaction with RAP70N. **A**. 50nmol of the indicated GST-tagged Tag peptides were separated by SDS-PAGE and detected with anti-GST (top) or pAb416 (bottom). **B**. Prior to mixing with GST-RPA70N, purified Tag was incubated with pAb416, non-immune mouse IgG, or in the absence of antibody as indicated. Bound proteins were eluted, separated by SDS-PAGE and detected with pAb101 (recognizes Tag) or anti-GST as indicated.

To provide additional evidence for the specificity of the binding interaction, we utilized a monoclonal antibody (pAb416) previously shown to recognize an epitope in the N-terminal portion of Tag [28]. We mapped the epitope for pAb416 to amino acids 84–130 of Tag (Fig. 3A). Since this epitope overlaps the RPA70N binding surface, we hypothesized that pAb416 might block the interaction between RAP70N and Tag. Full-length Tag was pre-incubated with control non-immune mouse IgG or pAb416 at a 1:1 molar ratio (Fig. 3B, lanes 3 and 5, respectively) or with no antibody (Fig. 3B, lane 4) prior to mixing with immobilized GST-RPA70N. As predicted, pre-incubation of Tag with pAb416 reduced the interaction of Tag with GST-RPA70N to below detection, while binding was retained upon treatment with the control IgG. Together, these results suggest that Tag residues 84–130 are both necessary and sufficient to mediate the RPA70N-Tag interaction *in vitro*.

### Tag(84–130) binds within the basic cleft of RPA70N

**Fig 4:**
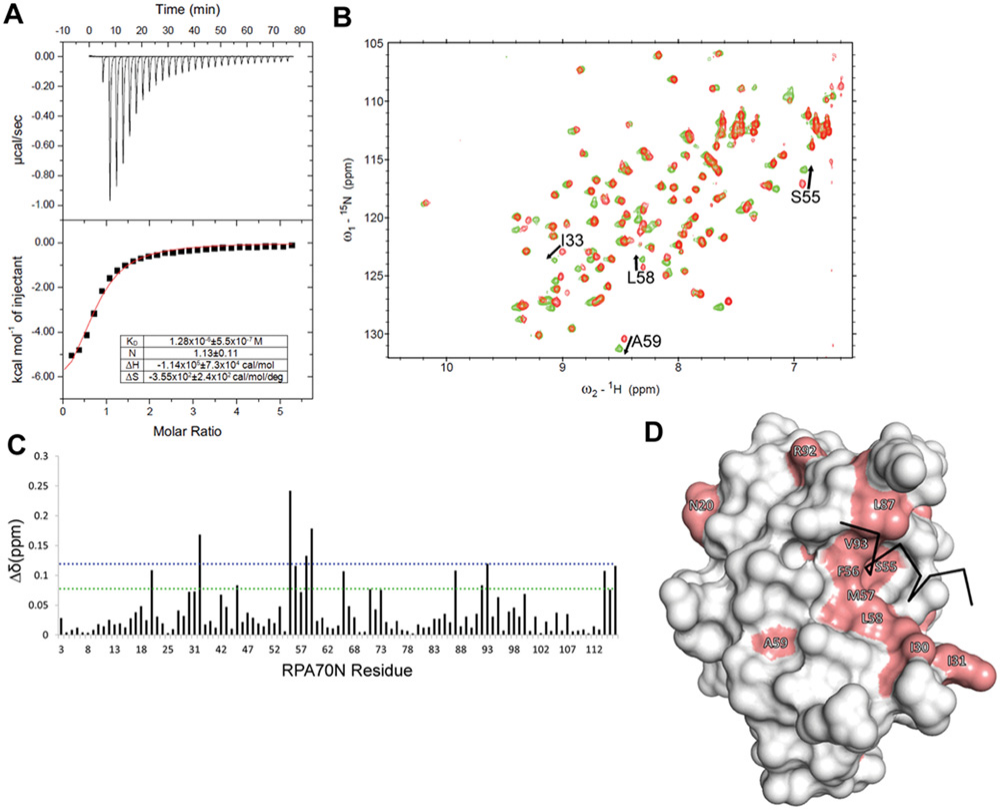
Tag(84–130) interacts with basic cleft of RPA70N. **A**. ITC raw heats and isothermogram for the titration of Tag(84–130) into RPA70N. **B**. ^15^N-^1^H HSQC NMR spectra of ^15^N-labeled RPA70N obtained in the absence (red) and presence (green) of Tag(84–130). The residues with the largest peak shifts are identified. **C**. Quantification of the chemical shift perturbations observed upon addition of Tag(84–130) to RPA70N. Dotted horizontal lines are drawn at one (green) and two (blue) standard deviations above the mean. **D**. Residues exhibiting chemical shift perturbations greater than one standard deviation above mean were mapped onto the crystal structure of RPA70N (Protein Data Bank accession no. 2B3G) in salmon. A black stick representation of the p53 peptide backbone from its RPA70N complex is shown to indicate its location within the basic cleft.

Isothermal titration calorimetry was used to determine the binding affinity of RPA70N for Tag(84–130). The binding isotherm for titration of purified Tag(84–130) into a solution of RPA70N was fit with a single-site binding model and provided a dissociation constant (K_d_) of 1.28 ± 0.5 μM (Fig. 4A). This affinity is similar to that of other proteins that bind to the RPA70N domain including HDHB and ATRIP [23, 31].

To identify the Tag binding site on RPA70N, we performed NMR chemical shift perturbation experiments using ^15^N-enriched RPA70N titrated with Tag(84–130). A series of ^15^N-^1^H HSQC spectra containing increasing concentrations of unlabeled Tag(84–130) revealed chemical shift perturbations for a select number of peaks in the spectrum (Fig. 4B and C), indicating binding to a specific site. To visualize the Tag(84–130) binding site, all RPA70N residues with a chemical shift perturbation greater than one standard deviation above the mean were mapped onto the structure (Fig. 4D). These results reveal that the primary binding surface is the common RPA70N basic cleft previously observed for p53, ATRIP, RAD9, and MRE11 [22, 24, 25].

To further confirm that Tag(84–130) binds in the RPA70N basic cleft, we repeated the pull-down experiments using a double charge reversal mutant (RPA70N R41E/R43E) in the basic cleft that had been shown previously to inhibit binding of target proteins to RPA70N [22]. Indeed, full-length Tag fused to GST is unable to pull down His-RPA70N R41/43E (Fig. 1C, lane 4). Similarly, His-RPA70N R41/43E protein fails to bind any portion of Tag (Fig. 2B, bottom panel). Thus, mutation of the two charged residues in the RPA70N basic cleft abolished the interaction with Tag. Together, these data show that the RPA70N-Tag interaction is specific and that Tag(84–130) binds to the basic cleft of RPA70N.

### Two acidic motifs in Tag(84–130) contribute to binding to RPA70N

**Fig 5:**
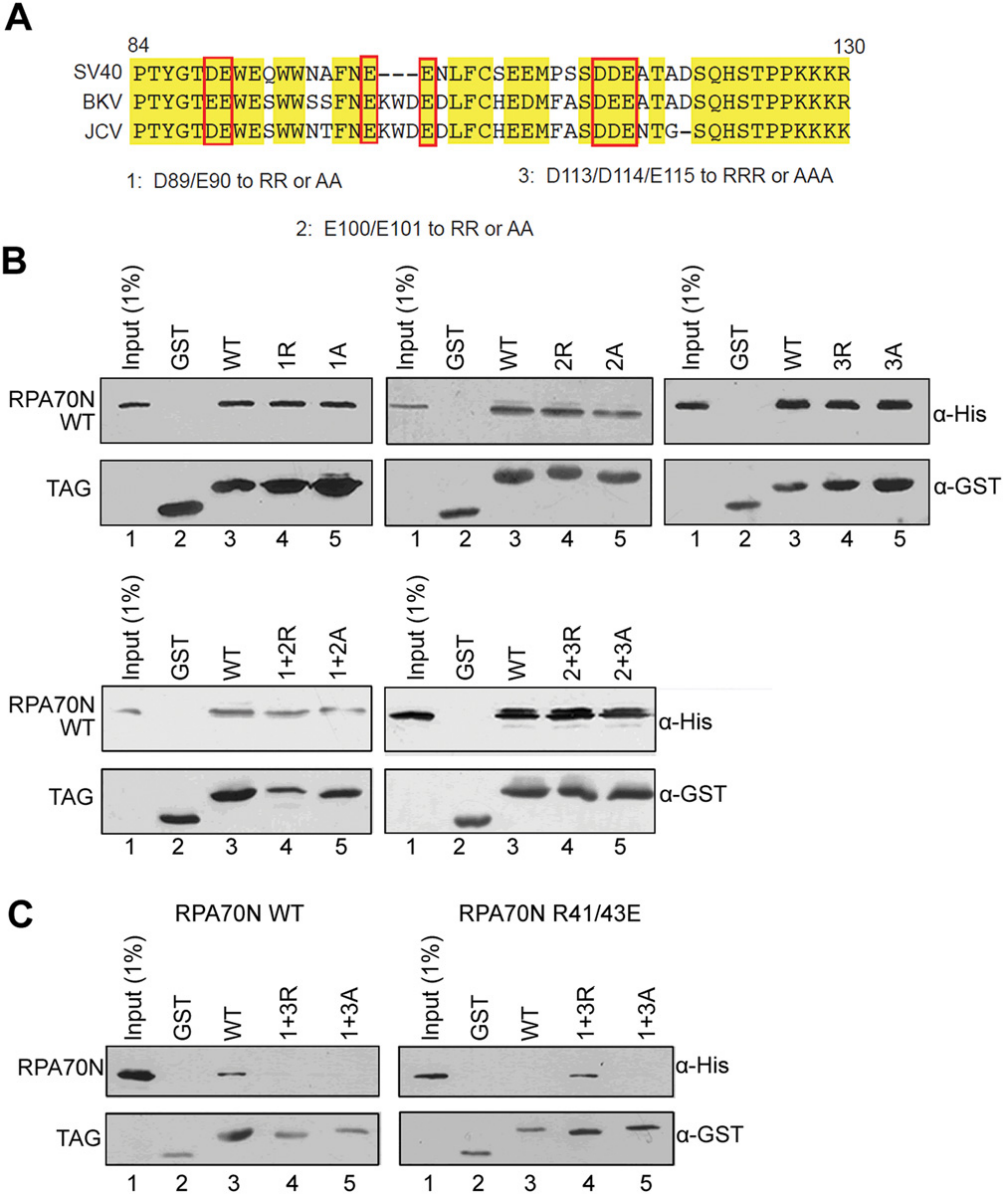
Two clusters of residues in Tag(84–130) contribute to the interaction with RPA70N. **A**. Sequence alignment of the alpha-4 helix and linker region of the large T antigen from SV40, BKV and JCV. The three clusters of residues targeted for mutagenesis are boxed in red. Residues in each cluster (1, 2 and 3) were mutated to either alanine (A) or arginine (R) in the context of Tag(84–130). Mutants in which amino acid changes in each cluster were combined (1+2, 2+3 and 1+3) were also created. (B) GST pull-down assays performed using WT or mutant His-RPA70N and GST-tagged Tag(84–130) as indicated. (**C**) GST pull-down assays performed with RPA70N WT (left) or RPA70N R41/43E (right) and Tag(84–130) with amino acids of cluster 1 and 3 mutated to either alanine or arginine as indicated.

Protein interactions in the basic cleft of RPA70N typically involve a contiguous segment of residues with acidic and aromatic character that undergo a disordered to ordered transition to an α-helical conformation [22, 24, 25]. Tag(84–130) contains three clusters of acidic residues that could fulfill these criteria and that are conserved in other polyomavirus Tag proteins (Fig. 5A). To test the contribution of these regions, we mutated the acidic residues boxed in Fig. 5A to either alanine or the basic amino acid arginine within the context of GST-Tag(84–130) and performed GST-pull down assays with His-RPA70N. Interaction of His-RPA70N with WT GST-Tag(84–130) was repeated in each panel for comparison. Individual mutation of each of the three clusters of basic residues did not perturb the association between RPA70N and Tag(84–130) (Fig. 5B). Likewise, combining the mutations in clusters 1 and 2 or 2 and 3 had no detectable effect on the interaction (Fig. 5B). However, making five alanine mutations of D89 and E90 in cluster 1 plus D113, D114, and E115 in cluster 3 (GST-Tag(84–130) 1+3A) did abolish the interaction with WT RPA70N (Fig. 5C, left panel). To further test the hypothesis of a significant electrostatic component to binding, we mutated the same five Tag residues to arginine (GST-Tag(84–130) 1+3R) and investigated binding to RPA70N WT and the RPA70N R41/43E mutant previously shown to inhibit binding to the basic cleft (Fig. 2B). Consistent with the hypothesis, we found that the combined five charge reversal mutations restored binding of Tag(84–130) to the RPA70N R41/43E mutant (Fig. 5C, right panel).

It is striking that two different clusters of amino acids within Tag(84–130) separated by >20 residues contribute to the interaction with RPA70N. Individually, each of these clusters resembles the checkpoint recruitment domain (CRD) found in other proteins such as ATRIP and RAD9 that bind to the RPA70N basic cleft [22]. The second cluster encompassing D113-E115 is located within the unstructured linker between the J-domain and the OBD of Tag and presumably folds upon binding as is the case for the p53 transactivation domain, ATRIP and other RPA70N interaction motifs [24]. The first cluster containing D89 and E90 is located at the N-terminus of the fourth helix of the Tag J-domain. This helix extends to N102. The fact that this sequence exists within a pre-formed helix presumably facilitates binding to RPA70N as it would not need to pay the entropic penalty for folding. The mutagenesis results (Fig. 5B) showing that substitutions in either cluster of acidic residues in Tag(84–130) alone are insufficient to abrogate binding to RPA70N is different from those described previously. For ATRIP, RAD9, MRE11 and HDHB, mutations of single clusters of acidic residues within the RPA70N binding motif eliminate binding [22, 24, 25]. Our observation for Tag(84–130) is surprising because RPA70N is unlikely to accommodate more than one helix at a time. One model that could explain the experimental observations is that the interaction is not mediated by a single helix and that the Tag motif is bidentate. In this model, the >20 residue separation in sequence between the key two acidic clusters would not correspond to significant spatial separation. In fact, there are 11 disordered residues between the D113-E115 cluster and the C-terminal end of the fourth helix at N102 [9, 32]. Hence, it is conceivable that this segment of the flexible LXCXE linker folds back upon itself, thereby positioning the two acidic clusters in close enough proximity for both to be engaged in the interaction with RPA70N. Further biochemical and structural analysis will be required to test this hypothesis and more specifically determine how these two clusters function together to mediate the interaction with RPA70N.

## Conclusion

Our studies bring the number of characterized interactions between RPA subunits and SV40 Tag to three. These multiple interaction surfaces may help coordinate the dynamic nature of the Tag-RPA interaction needed for successful SV40 replication. Alternatively, the interaction of Tag with the basic cleft of RPA70N opens the possibility that SV40 Tag competes with cellular factors for this binding site. Further studies are needed to establish the functional role of Tag interaction with RPA70N and understand how these interactions are coordinated to promote viral replication.
